# Prooxidant–antioxidant balance, hsTnI and hsCRP: mortality prediction in haemodialysis patients, two-year follow-up

**DOI:** 10.1080/0886022X.2017.1323645

**Published:** 2017-05-11

**Authors:** Tanja Antunovic, Aleksandra Stefanovic, Najdana Gligorovic Barhanovic, Milica Miljkovic, Danilo Radunovic, Jasmina Ivanisevic, Vladimir Prelevic, Nebojsa Bulatovic, Marina Ratkovic, Marina Stojanov

**Affiliations:** a Centre for Clinical-Laboratory Diagnostics, Clinical Centre of Montenegro, Podgorica, Montenegro;; b Department of Medical Biochemistry, University of Belgrade, Faculty of Pharmacy, Belgrade, Serbia;; c Clinic for Urology and Nephrology, Clinical Centre of Montenegro, Podgorica, Montenegro;; d Clinic for Cardiac Diseases, Clinical Centre of Montenegro, Podgorica, Montenegro;; e Faculty of Medicine, University of Montenegro, Podgorica, Montenegro

**Keywords:** Mortality, oxidative stress, prooxidant–antioxidant balance, haemodialysis, cardiovascular disease, inflammation

## Abstract

Oxidative stress and inflammation are highly intertwined pathophysiological processes. We analyzed the markers of these processes and high-sensitive troponin I (hsTnI) for mortality prediction in patients on haemodialysis. This study enrolled a total of 62 patients on regular haemodialysis. The patients were monitored for two years, and the observed outcomes were all-cause and cardiovascular mortality. Blood samples were taken before one dialysis session for analysis of the baseline concentrations of prooxidant–antioxidant balance (PAB), total antioxidant status (TAS), total oxidative status (TOS), hsTnI, hsCRP and resistin. The overall all-cause mortality was 37.1% and CVD mortality 16.1%. By univariate and multivariate logistic regression, our findings suggest that good predictors of all-cause mortality include hsCRP and PAB (*p* < .05) and of CVD mortality hsCRP (*p* < .05) and hsTnI (*p* < .001). To evaluate the relationship between the combined parameter measurements and all-cause/CVD mortality risk, patients were divided into three groups according to their PAB, hsCRP and hsTnI concentrations. The cutoffs for hsCRP and hsTnI and the median for PAB were used. Kaplan–Meier survival curves pointed out that the highest mortality risk of all-cause mortality was in the group with hsCRP levels above the cutoff and PAB levels above the median (*p* < .001). The highest risk of CVD mortality was found in the group with hsCRP and hsTnI levels above the cutoff levels (*p* = .001). Our data suggest that hsCRP and PAB are very good predictors of all-cause mortality. For CVD complications and mortality prediction in HD patients, the most sensitive parameters appear to be hsTnI and hsCRP.

## Introduction

Despite novel therapy management and intervention strategies, the poor survival rate of patients with end-stage renal disease (ESRD) on haemodialysis (HD) remains an issue of great concern worldwide. Since the major causes of death are cardiovascular diseases (CVD), different CVD risk biomarkers have been studied and many of them have been shown to be powerful predictors of mortality [1,2].

Oxidative stress can be considered as an imbalance in the reactive oxygen species (ROS) production/degradation ratio. Renal dysfunction is frequently associated with oxidative stress, since the levels of different markers like malondialdehyde and F2-isoprostanes are increased in patients with ESRD [3,4]. Additionally, in ESRD, a reduction in the plasma total antioxidant capacity (TAC) levels has been observed, probably due to malnutrition, a regular complication of this disease [5]. Oxidative stress in HD patients is usually assessed by measuring prooxidant levels, such as concentrations of malondialdehyde or hydroperoxides, or antioxidants like superoxide dismutase or myeloperoxidase activities. Prooxidant–antioxidant balance (PAB) is the only oxidative stress biomarker which measures prooxidants and antioxidants simultaneously in one sample, assessing their balance.

Cardiac troponin T (cTnT) and troponin I (cTnI) reflect ischemic cardiac damage, but in patients with severe renal disease, their levels are elevated without any evidence of acute coronary syndrome (ACS). Asymptomatic elevation of cardiac troponins in HD patients is possibly linked with oxidative stress and inflammation [6,7]. The elevation of cTnT and cTnI levels in HD patients corresponds with a 2- to 5-fold increase in mortality. The prevalence of cTnT increment in those patients is higher than that of cTnI [8,9]. Highly sensitive assays of cardiac troponins can detect even minor myocardial injury. Highly sensitive troponin T (hsTnT) has been studied more extensively as a mortality predictor in the HD population compared to troponin I (hsTnI).

Numerous studies have reported an association between markers of inflammation like CRP, IL-6, TNF-α and fibrinogen and chronic kidney disease (CKD) [10]. Moreover, it has been shown that elevated CRP, IL-6 and fibrinogen are independent predictors of cardiovascular outcomes in CKD patients [11].

It is known that oxidative stress subsequently causes inflammation which is the key factor of many human diseases including coronary diseases [12]. Oxidative stress and inflammation are highly intertwined processes in CKD, main causes of chronic kidney disease progression development and associated complications among which the central role has CVD [13].

Resistin is an adipocytokine and is known to be associated with inflammatory, metabolic and vascular abnormalities. The serum concentration of resistin is elevated in patients with end-stage renal disease, and there is a strong correlation between resistin and hsCRP concentrations and resistin and residual renal function in CKD patients [14,15]. High levels of resistin correlate with mortality in patients with coronary disease or acute myocardial infarction and the levels of this marker were predictors of atrial fibrillation in the Framingham Heart Study population. In addition, some authors have confirmed the significance of the serum resistin for mortality prediction in CKD patients [16,17].

The aim of this study is to evaluate the mortality risk predictive value of oxidative stress and inflammation biomarkers in haemodialysis patients. In the study, we have also included hsTnI, a marker of ischemic cardiac damage (Table 1).

## Methods

### Study design

This was a single-centre prospective study which enrolled patients on regular haemodialysis treatment at the Hemodialysis Centre of the Clinical Centre of Montenegro in Podgorica, Montenegro. The Ethics Committee of the Clinical Centre of Montenegro approved the study and all the patients gave written informed consent. Laboratory data were collected before one HD session at the study start, and then clinical status of the patients was monitored for two years (September 2013–September 2015). Our primary observed outcomes were all-cause mortality and mortality due to cardiovascular disease (CVD mortality).

### Patients and clinical data

The patients enrolled in this study were diagnosed with ESRD according to K/DOQI guidelines [18]. All the patients were on maintenance haemodialysis three times per week, with an average duration of single session being 4 h, using high-flux dialyzers with polysulfone membranes. The exclusion criteria were as follows: undergoing dialysis for less than 3 months, malignancy, acquired immunodeficiency, metabolic diseases, hepatic cirrhosis, patients on antiviral therapy and those taking antioxidants and vitamins, except vitamin D and folic acid, prior to dialysis. The prevalence of CVD in studied population was 39% with coronary heart disease and peripheral vascular disease being the most prevalent. Patients were not hospitalized in the previous two weeks and none had the symptoms of ACS. From all the patients, we obtained the relevant data (age, gender, weight, height, primary renal disease, duration of dialysis from the first session until the observed one, smoking habit, past medical history and current medications). The adequacy of the dialysis was determined by calculating the Kt/V and the urea reduction rate (URR). A Kt/V value of >1.2 and URR >65% were indicators of an adequate dialysis dose. Pre- and post-dialysis blood pressures (BPs) were measured for all the patients. Pre-dialysis BP was measured at the beginning of HD after 15 min at rest. Post-dialysis BP was measured at the end of HD, 15 min after disconnecting from the dialysis circuit. Body mass index (BMI) was calculated using the standard formula: body weight in kilograms/(body height in meters)^2^. The body weight in this formula was oedema-free mass, i.e. post-dialysis dry weight, according to recommendations [19].

### Blood samples and laboratory data

Blood samples were withdrawn before dialysis for measurement of the baseline concentration of the following biochemical parameters: creatinine, urea, uric acid and their associated markers: cholesterol, triglycerides, HDL cholesterol, LDL cholesterol, apoAI, apoB, hsCRP, resistin, prooxidant–antioxidant balance (PAB), total antioxidant status (TAS), total oxidative status (TOS) and hsTnI. Creatinine, urea, uric acid, cholesterol, triglycerides, HDL cholesterol and LDL cholesterol were measured on a Cobas c6000 (Roche Diagnostic, GmbH, Mannheim, Germany) by routine spectrophotometric methods; hsTnI on an Architect i2000 (Abbott Laboratories, Diagnostic Division, Abbott Park, IL); and apoA, apoB and hsCRP on a BNII nephelometer (Siemens Healthcare GmBH, Erlangen, Germany) by immunoassays. The prooxidant–antioxidant balance (PAB) was measured according to a previously published method by Almadari et al. [20] modificated in biochemical laboratory at Faculty of Pharmacy in Belgrade. Method principle is based on 3,3',5,5'-tetramethylbenzidine and its cation, which were used as redox indicators. They participate in two simultaneous reactions. Standard solutions were prepared by mixing varying proportions of 0–100% of 250 μmol/L hydrogen peroxide with 10 mmol/L uric acid. The intra-assay and inter-assay coefficients of variation were 6.6% and 7.2%, respectively. PAB values are expressed in arbitrary HK units, which correspond to the percentage of hydrogen peroxide in the standard solution. TOS was measured by the method of Erel et al. [21]. Serum TAS levels were determined by a novel automated method of Miller et al. using commercial kit (Randox Laboratories Limited, Crumlin County Antrim, UK) [22]. Resistin was estimated by Sandwich ELISA kit (R&D Systems, Inc., Minneapolis, MN) suitable for measuring natural and recombinant human resistin in most cell culture supernate, serum and plasma. The majority of the variables were determined on the day the blood samples were taken. The rest were stored at −80 °C.

### Statistical analysis

Continuous variables were expressed as mean ± SD or median with interquartile range, and categorical variables were expressed as a percentage. Comparisons between groups were analyzed using an independent *t-test* or a Mann–Whitney *U-test* for continuous variables and the Pearson Χ^2^test for categorical variables. Binary univariate logistic regression was used to identify the predictors of mortality in HD patients. Survivors in both groups were used as the reference groups and were coded 0. Non-survivors (all-cause) and non-survivors (CVD) were coded 1. For each hazard ratio (HR), we estimated two-tailed probability values and the 95% confidence interval (95% CI). To seek possible independent predictors of mortality in HD patients, we performed multiple logistic regression analysis where all significant predictors by univariate analysis adjusted for other variables with *p* < .1 in univariate analysis. Adjustment was performed to correct the possible interaction of investigated parameters. The Kaplan–Meier survival curve at 24 months was used to evaluate the difference between combined variables with significant ability for mortality prediction proven by logistic regression. Groups were compared using a log-rank test. All the statistical tests were two-sided, and differences were considered statistically significant where the *p*-value < .05.

## Results

A total of 62 patients (30 males and 32 females) with a mean age of 57.8 ± 10.2 years are enrolled in this study. The aetiologies of CKD are as follows: hypertension in 20 patients (32.3%), polycystic kidney disease in 12 patients (19.4%), chronic glomerulonephritis in 7 patients (11.3%), diabetes mellitus in 5 patients (8.1%), chronic pyelonephritis in 3 patients (4.8%), kidney stones in 3 patients (4.8%), Balkan endemic nephropathy in 2 patients (3.2%), other conditions in 7 patients (11.3%) and unknown cause in 3 patients (4.8%). After 2 years, the overall all-cause mortality was 37.1% (23 patients) and CVD mortality 16.1% (10 patients). In Table 2, the basic demographic characteristic and the routinely assessed baseline variables according to patient’s survival status are presented.

**Table 1.** Biomarkers studied in this paper.

Biomarker (abbreviation)	Biomarker (full name)	Biochemical significance	Summary of our findings
hsCRP	High-sensitive CRP	Inflammatory marker	Great predictor of mortality, all-cause and CVD
PAB	Prooxidant–antioxidant balance	Marker of both oxidative and antioxidative processes	Great predictor of all-cause mortality, in combination with CRP even better prediction possibility
TAS	Total antioxidative status	Measure of antioxidative protection	Significantly higher in survival group, no significance in mortality prediction
TOS	Total oxidative status	Marker of oxidative stress	No significance between survivors and non-survivors, no significance in mortality prediction
hsTnI	High-sensitive troponin I	Reflects ischemic damage of cardiomyocytes	Predictor of CVD mortality, the best prediction possibility when used with CRP
Resistin	Resistin	Inflammatory cytokine, link between inflammation with metabolic and vascular pathways	No significance between survivors and non-survivors, no significance in mortality prediction

There were no significant differences between the survival and both non-survival groups for age, sex, BMI and smoking habit. Regarding the adequacy of a single dialysis session, Kt/V and URR values were the highest among survivors, but there were no significant differences between the groups. Cholesterol, HDL cholesterol and LDL cholesterol were significantly higher among survivors compared to all-cause non-survivors. Univariate logistic regression shows a significant relationship between total cholesterol and LDL cholesterol in all-cause mortality (Table 3), but the significance disappeared in the multivariate model (Table 4). No significant difference was found between the groups for BMI. The creatinine level measured before the HD session was higher in the survival group compared to the non-survival ones. Table 5 describes the biochemical markers of inflammation, oxidative stress and hsTnI according to their survival status.

**Table 2.** Characteristics of patients according to their survival status.

	Survivors *n* = 39	Non-survivors all-cause *n* = 23	*p*-Value survivors, non-survivors all-cause	Non-survivors CVD *n* = 10	*p*-Value survivors, non-survivors CVD
Age, years	57.5 (50.25–63.0)	60.0 (55.0–68.5)	.077	56.0 (55.0–67.5)	.709
Sex	53.8% males(21)	39.1% males (9)	.393	60%males (6)	1
BMI, kg/m^2^	23.09 ± 3.27	23.48 ± 3.58	.631	23.86 ± 3.16	.363
Smokers	35.9%(14)	34.8% (8)	.862	20%(2)	.463
HD duration, days	1148 (471–2340.25)	1777.5 (425–3148)	.264	1656 (372.5–2401)	.778
Systolic before HD, mmHg	143.9 ± 21.8	139.5 ± 23.2	.465	148.3 ± 24.7	.385
Systolic after HD, mmHg	130.4 ± 20.4	127.7 ± 25.8	.661	135.6 ± 30.5	.511
Kt/V	1.16 (1.05–1.25)	1.10 (0.97–1.20)	.083	1.08 (1.01–1.14)	.281
URR	64.1 (58.8–68.4)	59.7 (53.2–67.1)	.124	57.8 (48.5–65.1)	.108
Urea before HD mmol/L	29.5 ± 5.9	30.9 ± 7.9	.433	34.8 ± 7.9	**.012**
Creatinine before HD, μmol/L	1038.8 ± 197.8	910.3 ± 198.4	**.016**	964.4 ± 208.8	.658
Uric before HD, μmol/L	412.1 (361.0–482.2)	411.5 (362.5–502.4)	.503	406.6 (369.3–479.3)	.826
Cholesterol, mmol/L	4.36 (3.67–4.72)	3.72 (3.06–4.36)	**.041**	4.03 (3.09–4.11)	.138
Triglycerides, mmol/L	1.43 ± 0.61	1.53 ± 0.68	.59	1.38 ± 0.42	.649
HDL cholesterol, mmol/L	0.86 (0.74–1.07)	0.73 (0.62–0.89)	**.025**	0.73 (0.61–0.81)	.065
LDL cholesterol, mmol/L	2.55 (2.12–3.02)	2.03 (1.92–2.65)	**.008**	2.03 (1.95–2.66)	.153
ApoAI, g/L	1.38 ± 0.23	1.27 ± 0.24	.089	1.24 ± 0.20	.135
ApoB, g/L	0.79 ± 0.17	0.80 ± 0.18	.742	0.83 ± 0.18	.443

Data are presented as Mean ± SD, Median (25–75 percentile) or percentage (count) and were compared by the Student?s t test or Mann-Whitney U nonparametric test, whereas categorical variables are presented as relative frequencies and were compared by the Chi-square test.

CVD: cardiovascular disease; BMI: body mass index; HD: hemodialysis; URR: urea reduction rate.

Bold values indicates statistically significant *p*-value < .05.

**Table 3.** Predictors of all-cause and CVD mortality: univariate logistic regression analysis (enter method).

	Mortality			
	All-cause		CVD	
Variables	HR (95% CI)^a^	*p*	HR (95% CI)^a^	*p*
Age, years	1.064 (1.012–1.118)	<.05^b^	1.051 (0.974–1.135)	.198
Smoking	1.01 (0.424–2.408)	.982	2.013 (0.418–9.691)	.383
BMI, kg/m^2^	1.017 (0.907–1.141)	.768	1.072 (0.893–1.286)	.457
TC, mmol/L	0.54 (0.319–0.917)	<.05^b^	0.541 (0.239–1.221)	.139
TG, mmol/L	1.158 (0.632–2.12)	.636	0.817 (0.239–2.285)	.700
LDL-C, mmol/L	0.372 (0.176–0.783)	<.05^b^	0.440 (0.140–1.389)	.162
HDL-C, mmol/L	0.341 (0.080–1.456)	.146	0.142 (0.015–1.327)	.087^b^
hsCRP, mg/L	1.021 (1.010–1.033)	<.001^b^	1.023 (1.005–1.048)	.054^b^
Resistin, mg/L	0.691 (1.93–2.481)	.571	1.283 (0.226–7.280)	.779
hsTnI, ng/L	1.007 (1.004–1.011)	<.001^b^	1.010 (1.004–1.016)	<.001^b^
TAS, mmol/L	0.061 (0.006–0.653)	<.05^b^	0.096 (0.003–1.746)	.171
TOS, μmol/L	1.006 (0.985–1.026)	.594	1.008 (0.981–1.036)	.551
PAB, HK units	1.035 (1.021–1.049)	<.05^b^	1.003 (1.012–1.055)	<.05^b^

^a^HR: hazard ratios; 95% CI: 95% confidence interval.

^b^Variables with *p* < .10 were entered in multivariate logistic regression analysis.

**Table 4.** Predictors of all-cause and CVD mortality: multivariate logistic analysis.

	Mortality			
	All-cause		CVD	
Variables	HR (95% CI)^a^	*p*^b^	HR (95% CI)	*p*^c^
Age, years	1.014 (0.944–1.088)	.707	–	
TC, mmol/L	2.046 (0.517–8.090)	.307	–	
LDL-C, mmol/L	0.168 (0.019–1.496)	.110	–	
HDL-C, mmol/L	–	–	0.029	.166
hsCRP, mg/L	1.085 (1.015–1.160)	**<.05**	1.076 (1.019–1.136)	**<.05**
hsTnI, ng/L	1.003 (0.997–1.009)	.340	1.013 (1.005–1.021)	**<.001**
TAS, mmol/L	0.027	.091	–	–
PAB, HK units	1.027 (1.001–1.054)	**<.05**	1.010 (0.976–1.045)	.566

^a^HR: hazard ratios; 95% CI: 95% confidence interval.

^b,c^ Values are adjusted for other variables with *p* < .10 in univariate logistic regression analysis.

Bold values indicates statistically significant *p*-value < .05.

**Table 5.** Characteristics of patients according to their survival status.

	Survivors *n* = 39	Non-survivors all-cause *n* = 23	*p*-Value survivors, non-survivors all-cause	Non-survivors CVD *n* = 10	*p*-Value survivors, non-survivors CVD
hsCRP, mg/L	2.59 (1.03–4.47)	12.20 (2.39–17.8)	**<.001**	13.7 (8.79–17.1)	**.001**
Resistin, mg/L	0.71 (0.52–1.03)	0.61 (0.50–0.75)	.386	0.72 (0.59–0.95)	.275
hsTnI, ng/L	12 (7–26)	38 (21.0–69.0)	**<.001**	40 (17.5–229.75)	**.039**
TAS, mmol/L	2.25 (2.11–2.32)	2.11 (2.04–2.20)	**.027**	2.12 (1.99–2.25)	.289
TOS, μmol/L	44.51 ± 24.43	48.87 ± 21.98	.545	50.52 ± 24.83	.52
PAB, HK units	14.86 (7.63–25.71)	39.66 (28.99–58.81)	**<.001**	42.13 (28.39–58.81)	**.013**

Data are presented as Mean ± SD, Median (25–75 percentile) and were compared by the Student?s t test or Mann-Whitney U nonparametric test.

CVD: cardiovascular disease; TAS: total antioxidant status; TOS: total oxidant status; PAB: prooxidant-antioxidant balance.

Bold values indicates statistically significant *p*-value < .05.

After a comparison of the HD survival group with the all-cause and CVD mortality, we concluded that there was a significant difference between survivors and all-cause non-survivors, and survivors and CVD non-survivors for the following parameters: hsCRP (*p* < .001, *p* = .001, respectively), hsTnI (*p* < .001, *p* = .039, respectively) and PAB (*p* < .001, *p* = .013, respectively). We also found significant difference for TAS but only between the survival and the all-cause mortality group (*p* = .027). hsCRP, hsTnI and PAB were all higher in the non-survival group. TAS levels were lower in the group of all-cause non-survivors compared to the survivors. In our study, resistin shows no significant differences between groups.

We used binary logistic regression to determine which of the investigated parameters had potential to predict all-cause and CVD mortality in HD patients. Unadjusted analysis indicated that age, TC, LDL-C, hsCRP, hsTnI, TAS and PAB concentrations were predictors of mortality in all-cause mortality group, while HDL-C, hsCRP, hsTnI and PAB concentrations were significant predictors in CVD mortality group (Table 3).

Thereafter, we constructed new logistic regression models to further test the potential independent predictive power of significant predictors by univariant analysis (Table 4). After adjustments, hsCRP and PAB concentrations remained significant independent predictors of mortality in the all-cause mortality group. In CVD mortality group, hsCRP and hsTnI continued to be significant predictors.

In order to evaluate the relationship between combined parameter concentrations and all-cause and CVD mortality risk, we divided the patients into three groups according to their PAB, hsCRP and hsTnI concentrations (parameters that showed significant predictive power by multivariate analysis). We have used cutoffs for hsCRP of 5 mg/L for all-cause and 3 mg/L for CVD mortality. The American Heart Association and the Centres for Disease Control and Prevention (AHA/CDC) have issued guideline for the utility of hsCRP in the primary prevention setting and in patients with ACS. The guideline recommended that a hsCRP concentration higher than 3 mg/L should be considered as a high risk for CVD development [23]. Also, hsCRP concentrations higher than 5 mg/L referred some inflammation process in organism, so we used this cutoff for all-cause mortality. For PAB, the median of values from our study was used as cutoff value for Kaplan–Meier survival curve constructions. As cutoff value for hsTnI, we used 26.2 ng/L that is the 99th percentile value, recommended by manufacturer (Architect STAT High Sensitive Troponin-I, Abbott Laboratories, Diagnostic Division, Abbott Park, IL).

**Figure 1.**
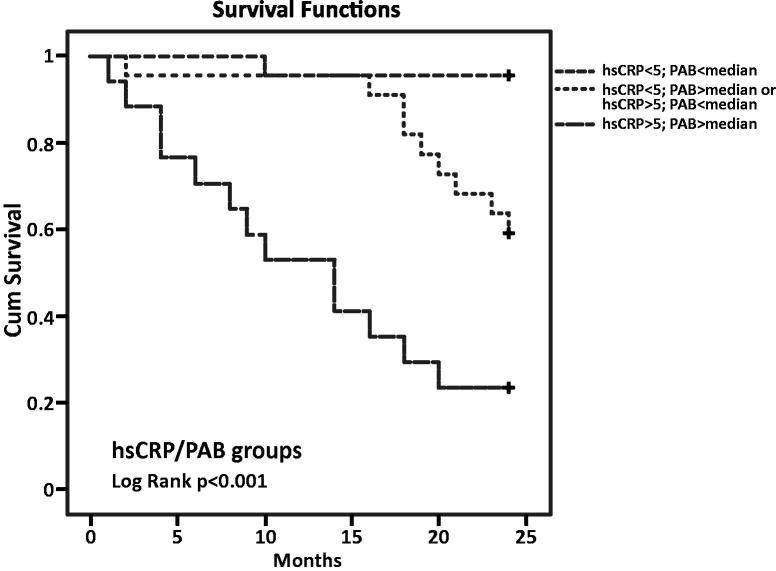
Kaplan–Meier survival curves by hsCRP and PAB groups (all-cause mortality).

In the all-cause mortality group were 23 patients with CRP values below 5 mg/L and PAB lower than the median, 22 patients with CRP or PAB higher than the cutoffs/median and 17 patients with PAB and CRP above the median/cutoffs. According to the Kaplan–Meier survival curves, the highest mortality risk for all-cause mortality was in the group with hsCRP levels above the cutoff and PAB levels above the median (*p* < .001). There was one deceased patient in the first group, nine in the second and 13 patients in the third group, respectively (Figure 1).

**Figure 2.**
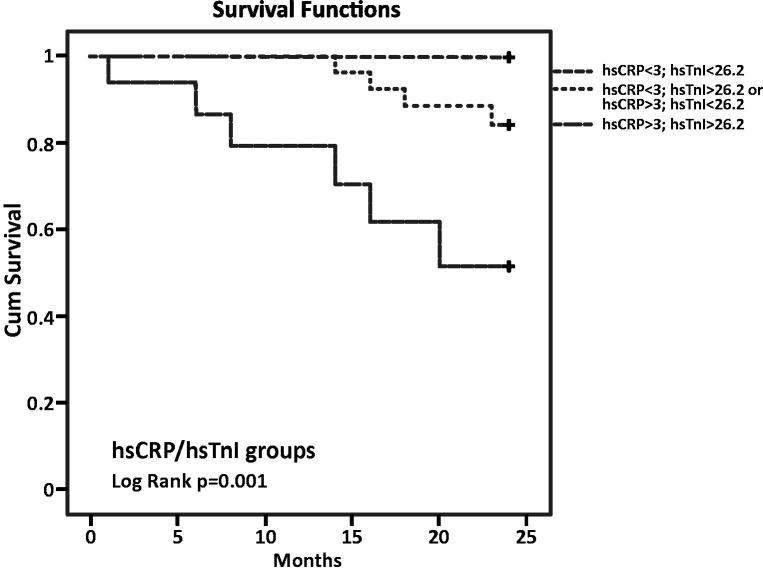
Kaplan–Meier survival curves by hsCRP and hsTnI groups (CVD mortality).

For CVD mortality assessment, we had three groups with 19, 27 and 16 patients. None of the patients from the first group with hsCRP and hsTnI concentrations lower than the cutoffs had cardiovascular complication over a 2-year period. However, four patients in the group with elevated hsCRP or hsTnI and six patients in the group with both markers above the cutoff died from a cardiovascular cause. The highest risk for cardiovascular deaths was related to hsCRP and hsTnI levels above the cutoffs (*p* = .001) (Figure 2).

## Discussion

The main goal of our study was to assess the potential significance of serum inflammatory markers, markers of oxidative stress and antioxidant protection as well as hsTnI in the mortality prediction of ESRD patients on haemodialysis.

There were no significant differences between groups for age, sex, smoking habit and systolic blood pressure. Although the lowest value of the calculated BMI levels was in the group of survival patients (23.09 ± 3.27), there were no significant differences between the groups. The adequacy of a single dialysis session and the duration of HD treatments in days from the first session to the observed one were also not significant. HDL cholesterol in our study was higher in the survival compared to the all-cause non-survival group. Total cholesterol and LDL cholesterol were also significantly higher among survivors compared to all-cause non-survivors. These two findings speak in favour of ‘reverse epidemiology’ in CKD patients. Some studies have suggested that not only total hypercholesterolemia but also LDL hypercholesterolemia tends to show a paradoxical association with better survival [24]. On the other hand, patients with CKD are at very high risk of developing CVD, and the recommended LDL cholesterol plasma value for them is below 1.8 mmol/L [25]. In our study, only 13.3% of patients had target LDL cholesterol values. Total cholesterol and LDL cholesterol showed a significant relationship with all-cause mortality using univariate logistic regression (Table 3), but that significance disappeared in the multivariate model (Table 4).

Resistin was included in the study because of its strong relationship with inflammation, vascular pathology and mortality prediction in patients with coronary heart disease, but our data could not prove the significance of resistin in clinical outcome of HD patients.

We investigated the mortality prediction value of three oxidative stress biomarkers and concluded that TAS was significantly lower in the all-cause mortality group compared to survivals but without significance in the CVD mortality group. One cause of decreased antioxidant capacity in HD patients is the loss of antioxidants via the dialysis process [26]. On the other hand, TOS was higher in both non-survival groups, but without significant differences between the groups. The PAB assay can measure simultaneously the prooxidant burden and the antioxidant capacity in one assay, giving thereby a redox index [27]. In our study, PAB was demonstrated to be the best marker of increased oxidative stress and the high impact of oxidative stress on mortality in maintenance haemodialysis patients. Univariate logistic regression for PAB in all-cause and CVD mortality groups showed the significance for both (*p* < .05) but after multivariate logistic regression analysis that significance remained only for all-cause mortality (*p* < .05). In summary, the role of oxidative stress in renal dysfunction is often underappreciated in clinical practice, but emerging evidence continues to highlight its strong correlation with cardiovascular morbidity and mortality [28]. Kaplan–Meier survival analysis for the combination of the hsCRP cutoff value (5 mg/L) and the median of the PAB value showed that the highest risk of all-cause mortality was in the group of patients with hsCRP values above the cutoff and PAB values above the median. There were 17 patients in that group and 13 of them diseased. Many oxidative stress biomarkers have been evaluated in different studies on haemodialysis patients, but we have demonstrated that the lesser-used PAB is a very powerful marker of oxidative stress and it should be a biomarker of interest in the future studies.

In our study, more than half of the patients (56.5%) had hsCRP levels above 3 mg/L, and 39.7% patients had hsCRP levels above the cutoff for infections in our hospital (5 mg/L), although in the survival group the median of hsCRP was 2.59 mg/L with an interquartile range 1.03–4.47 mg/L. Univariate and multivariate logistic regression of our data indicated that hsCRP is a strong predictor of mortality (both all-cause and CVD). Our findings are similar to many other studies conducted on larger groups of HD patients [29,30].

The hsTnI median in our entire cohort was 21 ng/L with interquartile range 8.75–39.75 ng/L. The overall 99th percentile cutoff value recommended by manufacturer is 26.2 ng/L. All our patients were asymptomatic regarding the presence of ACS but 38.7% of them had hsTnI concentration above the cutoff. The high prevalence of persistently elevated troponin levels in these patients may reduce the diagnostic specificity of troponin for AMI [31,32]. It is well known that cardiac troponin levels are higher in patients on haemodialysis compared to the general population, and the use of high-sensitivity assays, which by definition can detect cardiac troponins in ∼50–90% of healthy individuals [33], increased even more the number of HD patients with elevated levels of hsTnI or hsTnT. The median of values in the survival group of our study was 12 ng/L (interquartile range 7–26 ng/L) which is below the cutoff, but the medians in both non-survival groups were higher and univariate logistic regression analysis shows that hsTnI is a very good prognostic marker for all-cause and CVD mortality among patients on dialysis, while multivariate analysis proved that the statement is only for CVD mortality. Our results suggest that patients on haemodialysis with a lack of ACS symptoms and baseline values of hsTnI which are above the available cutoff have an increased risk of death caused by cardiovascular diseases. Alam et al. have also suggested an association between elevated troponin I levels and CVD mortality [34]. Kaplan–Meier survival analysis for the combination of cutoff values for hsCRP and hsTnI showed that the highest risk of CVD mortality was found in patients with hsCRP and hsTnI levels above the cutoff. Of our 10 deceased patients, six were in that group and none of them was in the group with hsCRP and hsTnI levels below the cutoff.

Reduction in oxidative stress will lead to improve renal function in CKD patients, and also it will lower inflammation and CVD risk [12,35]. There are many studies on treatments targeting oxidative stress in patients with renal dysfunction. The suggested therapeutic interventions aiming to reduce oxidative stress in HD patients include biocompatible membranes, the administration of antioxidants and substances indirectly affecting oxidative stress [36]. In terms of antioxidant vitamins, the role of vitamin C is controversial, but vitamin E has been shown to improve the oxidant/antioxidant imbalance in this group of patients. Antioxidant-based dialysis membranes, such as vitamin E-modified cellulose membranes to take one example, have been shown to improve endothelial dysfunction and reduce free radical production [28,37]. Omega-3 polyunsaturated fatty acids, N-acetyl cysteine, coenzyme Q_10,_ mitochondrial-targeted antioxidants (mitochondrial-targeted derivative of endogenous Q_10_, mitochondrial-targeted vitamin E) and carnitines have promising potential for future therapeutic approach in CKD patients, but multidrug therapy, targeting many pathways modifying by oxidant processes, will be very likely needed [35].

The limitation of our study is small sample size. Our study was done in one centre in country with around 600,000 inhabitants. Larger multicentre studies are needed for proving our data. Also we did not use any therapeutic interventions using antioxidants and that approach would be, in our opinion, very beneficial. The main reason for doing so was number of patients on HD treatment and impossibility to divide them into two groups (therapeutic and control). In our study, we prove enormous potential of PAB in oxidative stress–antioxidative protection evaluation and mortality prediction. Oxidative stress is still very difficult to measure and monitor because of large number of markers, many of them leaded to opposite study results and none of them standardized. PAB was not studied that much and that could be weak point of this study by some researchers. We, on the contrary, think that this is also the benefit of this study, because this marker has to be examined by much more researchers for definitely proving its potential.

## Conclusions

Our data suggest that patients decease after two years were more exposed to inflammation and oxidative stress compared to the survivors. PAB has shown significant potential for mortality prediction in CKD patients. Biomarkers of inflammation (hsCRP), oxidative stress (PAB) and hsTnI have demonstrated that they are very good predictors of all-cause and CVD mortality. The combination of hsCRP and PAB values proved to be a good tool for the assessment of all-cause mortality prediction, while hsCRP and hsTnI assayed together might target HD patients with a higher risk of developing CVD complications and death.
